# Imaging Modalities and Their Findings in Patients With Complex Regional Pain Syndrome: A Review

**DOI:** 10.7759/cureus.41747

**Published:** 2023-07-11

**Authors:** Adarsh Vardhan Tangella

**Affiliations:** 1 Internal Medicine, King George Hospital and Andhra Medical College, Visakhapatnam, IND

**Keywords:** fmri, diagnosis, imaging, chronic pain, crps

## Abstract

Complex regional pain syndrome (CRPS) is a systemic or regional pain pathology associated with the nondermatomal or dermatomal distribution of excruciating intolerable pain, which might be triggered by an insignificant or weak stimulus or sometimes without any. Its symptoms encompass neurological, musculoskeletal, dermatological, and vascular realms. It is usually preceded by an episode of nerve injury or intervention set in numerous circumstances ranging from trauma to surgeries to chronic diseases. CRPS has been shrouded in a veil of *mystery* and was called a *psychological* phenomenon without any proper organic basis when it was described by Ambroise Pare initially. This led to disproportionately fewer research investments into this disease. Given the great advancement of diagnostic modalities since its inception, researchers and physicians have been trying to identify the physiological basis for it and have succeeded. Numerous pathophysiological pathways have been involved in this disease, but all of them point toward the possibility of improper pain processing at various levels of the pain pathway along with brain plasticity leading to aberrant neuronal circuitry between different segments of the sensory cortex, basal ganglia, prefrontal cortex, and insula. This paper explores the various studies done to evaluate the role of different imaging modalities, ranging from three-phase bone scintigraphy (TPBS) to diffusion traction imaging (DTI).

## Introduction and background

Pathophysiology of complex regional pain syndrome

Complex regional pain syndrome (CRPS) has a complex pathophysiology, but the beginnings of the disease are rooted in a lot of common triggers [1]. Some of the most common triggers studied are trauma and surgeries [2]. Rarely, certain diseases such as malignancies can also trigger CRPS [3]. Typically, CRPS begins as a locoregional disease but has a chronically progressive timeline, making it a debilitating disease for patients. It ruins their quality of life and sometimes might also cause physical disability [1,2]. Inflammation and autoimmunity form a very important part of the pathophysiology of this disease [4]. 

Neuronal Autoimmunity

The role of autoantibodies in CRPS has been studied for several years now [5,6,7]. According to research done by Kohr et al. [5], about 90% of the cohort of adult CRPS patients had one of the two types of antibodies against the autonomic nervous system (ANS). The two common types include anti-beta-2 receptor antibodies and anti-muscarinic-2 receptor antibodies. About 55% of the cohort had both these antibodies in circulation [5]. This establishes the role of neuronal autoimmunity in patients with CRPS. It is also important to note that these autonomic receptors are present across a wide range of tissues - especially in the central nervous system. Beta-2 adrenergic receptors are present in the cerebellum, reticular formation, sympathetic ANS, astrocytes, and microglia, and muscarinic type 2 receptors are present in parasympathetic ANS, heart, pyramidal pathway, skeletal muscles, motor cortex, thalamus, and peripheral nerves. This can prove the reason for the wide spectrum of symptoms ranging from severe pain to movement disorders seen in patients with CRPS. An important question that arises from this discussion is about the source of these antibodies. The possible triggers can be due to past history of trauma or episodes of acute infarction such as stroke or myocardial infarction, which lead to the destruction of tissue and exposure of concealed receptor antigens, resulting in the production of autoantibodies targeting them.

Neuroinflammation

**Figure 1:**
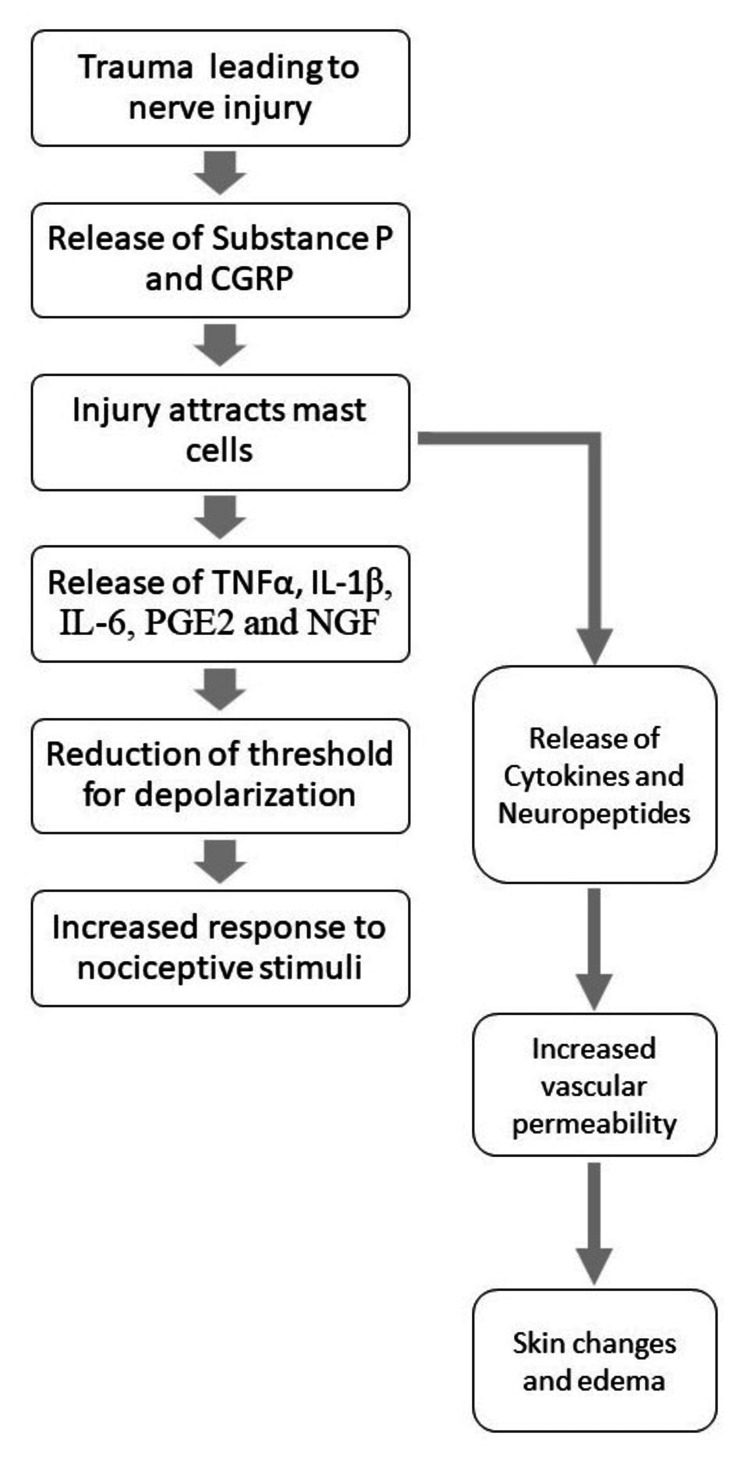
Role of neuronal inflammation in complex regional pain syndrome. Figure credits: Adarsh Vardhan Tangella. CGRP, calcitonin gene-related peptide; IL-1beta, interleukin 1 beta; PGE2, prostaglandin E2; NGF, nerve growth factor; TNF alpha, tumor necrosis factor alpha

Trauma is associated with varying degrees of peripheral nerve damage, depending on the extent and site of injury [4]. There is also associated sympathetic dysregulation due to inflammatory changes in the tracts [4]. Figure 1 shows the mechanism by which trauma-induced neuroinflammation can result in abnormally magnified pain sensations.

Post-traumatic Neuroinflammatory Tracks

Researchers have been pondering the question of the involvement of the central nervous system in CRPS and the evidence that the thalamus can be involved solidified the basis for CRPS being a CNS pathology. Studies done by Banati et al. have proved the existence of neuroinflammatory tracks where there is a slow ascent of inflammation beginning from the peripheral nerves, transmitted eventually via the spinal roots to the ascending tracts and finally to the thalamus. This is essentially mediated by the release of certain forms of cytokines at the neuronal terminals which in turn induce inflammation at the cell body of the subsequent neuron. It can also be associated with transient or permanent lowering of action potential thresholds which can lead to magnified signals with minimal stimulus [8,9,10]. The fact that the thalamus or the CNS at large can be involved might also be secondary to transient leakages in the blood-brain barrier [11]. These leaks might facilitate the entry of autoantibodies against the ANS to enter and attack various regions of the brain, resulting in subsequent symptoms depending on the site of the insult [11].

All these changes point toward the bigger question: Can these CNS changes be seen on imaging? Is it possible to diagnose CRPS and differentiate it from other forms of chronic pain such as neuropathies or fibromyalgia? It is interesting to note that there have been several studies that have shown a wide spectrum of abnormalities in imaging studies. The idea of this review is to highlight the findings observed in various radiological investigations performed.

The Spectrum of Symptoms and Criteria for Diagnosis

CRPS can sound like a misnomer when a wide range of patients are taken into consideration because, despite starting in a localized area, CRPS progressively worsens in the majority of the cases to involve multiple areas, which might or might not be related anatomically or neurologically. Given how this disease is not localized in the majority of the cases, it is important to note that there is a high probability of it being a disease involving the central nervous system and the peripheral nervous system.

The Budapest criteria have been used by physicians across the world to diagnose CRPS and have proven to have high sensitivity and specificity [12]. Although its usage has certain limitations, especially in cases of post-stroke CRPS [13], the majority agree that it established a certain diagnostic standard for confirmation of CRPS. It has four distinct domains across which signs and symptoms are assessed, and if the patient has satisfied at least one domain, they can be diagnosed with CRPS. Table 1 describes the domains of Budapest criteria. They have to be read in the context of continuous pain, which is disproportionate to any inciting event.

**Table 1:** Budapest criteria for the diagnosis of CRPS. Source: [13]. CRPS, complex regional pain syndrome

Signs (Must display at least one sign at the time of evaluation in two or more of the following categories)	Symptoms (Must report at least one symptom in three out of the four following categories)
Sensory: Evidence of hyperalgesia (to pinprick) and/or allodynia (to light touch and/or deep somatic pressure and/or joint movement)	Sensory: Reports of hyperalgesia and/or allodynia
Vasomotor: Evidence of temperature asymmetry and/or skin color changes and/or asymmetry	Vasomotor: Reports of temperature asymmetry and/or skin color changes and/or asymmetry
Sudomotor/Edema: Evidence of edema and/or sweating changes and/or sweating asymmetry	Sudomotor/Edema: Reports of edema and/or sweating changes and/or sweating asymmetry
Motor/Trophic: Evidence of reduced range of motion and/or motor dysfunction (weakness, tremor, and dystonia) and/or trophic changes (hair/nails/skin)	Motor/Trophic: Reports of reduced range of motion and/or motor dysfunction (weakness, tremor, and dystonia) and/or trophic changes (hair/nails/skin)

The main idea of this review is to understand the spectrum of radiological findings in patients with CRPS. A wide range of investigative studies have been performed to understand the neuronal circuitry, gray and white matter changes, and trophic and bone matrix-related changes. The methods that would be dealt with would be magnetic resonance imaging (MRI), functional MRI (fMRI) (with or without voxel-based morphometry), three-phase bone scintigraphy (TPBS), computed tomography (CT) scan, positron emission tomography-CT (PET-CT), and diffusion tensor imaging (DTI).

## Review

Role of fMRI and structural MRI (sMRI) in CRPS

Abnormal Brain Responses to Action Observation in CRPS

Patients had a significant association between movement-related pain and the visualization of the affected limb. When a smaller image of the limb was displayed, the intensity of pain and swelling was reduced compared to when a larger image of the limb was presented. This effect was particularly pronounced when the pain and swelling were more severe than usual. This shows that the changes in pain processing pathways become more centrally influenced and are seriously disrupted, resulting in the formation of aberrant neuronal connections as a consequence of constant background pain [14].

Crossroads Between Motor and Pain Pathways: The Role of Putamen in CRPS

Nonmotor basal ganglia connections are responsible for processing pain, sensory integration, visual processing, cognition, and emotion. Infraslow oscillations (ISO) are basal changes in frequency observed on an fMRI, which might be responsible for regulating behavioral performance and the probability of having seizures [15]. In a study by Lee et al., when ISO in basal ganglia was studied on fMRI sequences, CRPS subjects displayed increased ISO power in the putamen located contralateral to the affected limb - specifically involving the supplementary motor area hand, motor hand, and motor tongue. As an indication for possible aberrant neuronal circuitry secondary to chronic pain, resting connectivity between the aforementioned putaminal areas and projections from the caudate nucleus to cortical areas such as the primary motor cortex, supplementary and cingulate motor areas, parietal association areas, and the orbitofrontal cortex were enhanced when compared to controls. There was also a direct association between the degree of increase in ISO in the putamen and the degree of disability in the person. There was a significantly increased putamen - M1 (primary motor cortex) connectivity, which also translated to poorer motor performance, specifically in tasks involving faster and more accurate motor coordination [16]. The increased ISO is probably a result of astrogliosis as a consequence of constant pain stimulation, which leads to elevated astrocyte calcium levels and gliotransmitter release, resulting in an elevated oscillatory frequency [16]. Surprisingly, in the setting of elevated neuronal connectivity in the putaminal region, a bilateral reduction in the putaminal volume was observed in a study by Azqueta-Gavaldon et al. [17], which might be an indicator of poor motor function. Similar findings have also been observed in Parkinson’s disease [18]. Another crucial observation in this study was the altered relation between the degree of putaminal functional connectivity with pre-/postcentral gyrus and pain intensity. In healthy individuals who have been subjected to pressure pain constantly, there was a negative correlation between the above two parameters [19], but in patients with CRPS, there was a positive correlation between them [17].

Centralization of Effects of Chronic Neuropathic Pain in CRPS

Central sensitization, as observed in other chronic pain disorders such as fibromyalgia, also plays a key role in the pathophysiology of CRPS. A voxel-by-voxel analysis of fMRI sequences done by Di Pietro et al. showed enhanced thalamocortical neuronal connectivity - specifically to the contralateral orbitofrontal cortex, ipsilateral insula, bilateral amygdala, bilateral posterior parietal, cingulate, and primary somatosensory cortices (S1). There was a significant increase in the thalamus-S1 connectivity in CRPS subjects, which was also associated with reduced tactile discrimination of the painful hand. Reduction in thalamic blood flow and functional cortical reorganization in the primary somatosensory cortex (S1) are some of the most consistent findings [20].

Studying Effects of CRPS on Emotional and Autonomic Regions Using a Combination of fMRI and DTI

Patients with CRPS have a wide spectrum of presentations and emotional volatility, and higher incidence rates of depression or anxiety are usually seen in patients with chronic pain. This is usually a consequence of background pain stimulus-mediated alterations in the mesolimbic and mesocortical dopaminergic pathways and the primary focus of this neurochemical alteration is the midbrain [21]. But in CRPS, studies have shown that there are alterations in the neuronal circuitry between various cortical emotional areas, which can lead to excess or underexpression of them. One of them is a study by Geha et al. [22]. The basis for this study comes from an observation made by Apkarian et al. and Bechara that patients with CRPS patients have a worse emotional decision-making capacity when compared to other chronic pain patients, and this matches with patients who have lesions in the ventromedial prefrontal cortex (VMPFC) [23,24]. Geha et al. also hypothesized that since CRPS also has an autonomic dysregulation component, there is a probability of observing alterations in the autonomic outflow areas of the cortex, namely, VMPFC, anterior portion of the insula (AI), and dorsal anterior cingulate (also ACC) [22]. A key finding of their study was a generalized disruption in the gray-white matter relationship, which shows how damaging chronic pain can be in the long term. There was a significant negative correlation noted between the gray matter densities of VMPFC and AI and pain intensity and duration. The higher the pain, the lower the gray matter densities noted in these two areas. On DTI, the white matter connections from VMPFC in CRPS patients were variable where higher connectivity was observed with the insula and lower to the basal ganglia. Contralateral connectivity was also enhanced from VMPFC but not in the case of AI. There was also increased connectivity between VMPFC and nucleus accumbens (NAc), and the amount of this increase correlated positively with anxiety scores. The gray matter atrophy in VMPFC and AI correlates with the constant negative emotional state and autonomic dysfunction seen in CRPS due to the association of these areas with emotional control and reactivity to sensory stimuli, resulting in inappropriately exaggerated emotions such as anger but social-emotional stunting [22].

A voxel-based morphometry meta-analysis performed by Ma et al. [25], contrary to certain observations in previous studies, there was an increase in the gray matter volume (GMV) in the medial left superior frontal gyrus (SFGmedial) and left striatum but reduced GMV in the corpus callosum. The GMV changes in SFGmedial were inversely related to the disease duration. An important conclusion from this study was that this increased GMV in SFGmedial could be a functional compensation in CRPS patients. SFGmedial is associated with numerous neuropsychological disorders such as schizophrenia, obsessive-compulsive disorder, attention-deficit hyperactivity disorder, and bipolar disorder [26,27,28]. In a study by Barad et al. [29] where CRPS patients were compared to healthy controls, similar to the meta-analysis, there was an increase in GMV in the left dorsal putamen and the hypothalamus but lesser GMV in the cingulate, orbital frontal cortex, and left posterior insular region. The duration was associated inversely with GMV in the left dorsolateral prefrontal cortex (DLPFC). An important observation here is the GMV alteration in the putamen and the recognition of its significance. Putamen is known to be associated with somatic pain processing to a certain extent and is also known to have a somatotopic representation of bodily pain [30]. Activation of dopaminergic neurons in this region of the putamen is associated with reduced pain sensitivity, and hence, the increase in GMV here might be a compensatory mechanism to reduce the nociceptive inputs associated with CRPS [31]. Another crucial observation in this study is the atrophy of DLPFC. Although not central to the CRPS pathophysiology, transcranial magnetic stimulation of DLPFC has shown a certain degree of analgesic benefit [32], thereby pointing toward the hypothesis that this atrophy in DLPFC might be a consequence of an adaptive mechanism to reduce the effects of nociceptive inputs.

In a review by Thorp et al., it has been described that there has been an observed increase in functional connectivity of the posterior cingulate cortex (PCC) with the sensorimotor cortex, prefrontal cortex, posterior parietal cortex, inferior parietal lobule, thalamus, and anterior cingulate cortex (ACC) [33]. There is also a negative correlation between the functional connectivity between the sensorimotor cortex and DLPFC [33].

Role of CAT in CRPS

High-Resolution Peripheral Quantitative CT (HR-pQCT) for the Assessment of Bone Microstructure in CRPS

**Figure 2:**
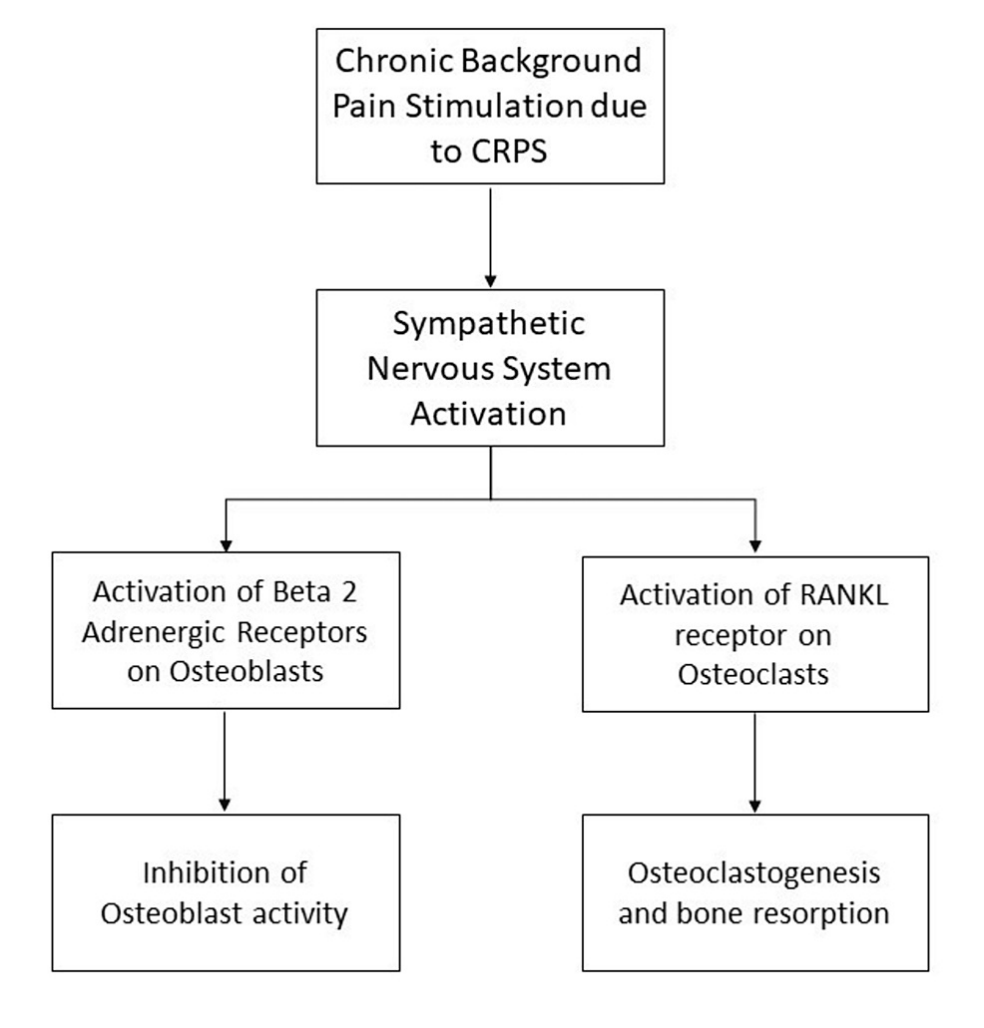
Mechanism of bone resorption and impaired mineralization in CRPS. Image credit: Adarsh Vardhan Tangella. CRPS, complex regional pain syndrome; RANKL, receptor activator of nuclear factor kappa-Β ligand

The endplate of the radius was used as a reference point for the assessment of the trabecular microstructure. Parameters used were trabecular bone volume to total bone volume (TV/BV), trabecular number (TbN), trabecular thickness (TbTh), cortical thickness (CTh), and cortical bone mineral density (C-BMD). The CRPS-affected limb had significantly lower TbN but higher TbTh. As time since the onset of the disease increased, TbN continued to decrease and TbTh continued to increase. This inverse relation between TbN and TbTh seems to be an adaptation to ensure that trabecular bone volume is maintained relative to total bone volume. These changes are said to be a consequence of a cascade of neuroendocrine-mediated pathological processes due to constant pain stimulation primarily mediated by the activation of osteoclasts and osteoblasts [34]. Figure 2 shows how CRPS causes the aforementioned changes.

Role of PET-CT in CRPS

CRPS has been known to cause neurotransmitter imbalances as a consequence of chronic background pain stimulation. Chronic pain is associated with neuroinflammation as mentioned earlier, leading to glial cell activation. Glial cell activation is a coping mechanism that is usually turned on when there is undue stress. But chronic glial cell activation is deleterious to the brain as microglial activation is associated with inflammatory reactions [35]. Studies on how 11 carbon-R-(2 chloro-phenyl)-N-methyl-N (1-methyl propyl)-3-isoquinoline-carboxamide PK11195 PET (11C-R-PK11195 PET) for CRPS are based on this principle of glial cell activation [33]. Activated glial cells express a translocator protein called TSPO for which 11C-(R)-PK11195 is a specific ligand [36]. A study conducted by Jeon et al. showed that there is a significantly higher 11C-(R)-PK11195 distribution volume ratio (DVR) in the caudate nucleus, putamen, NAc, and thalamus in CRPS patients when compared to healthy controls [37]. There was also a positive correlation between the intensity of pain and DVR of the caudate nucleus in CRPS patients [37]. These findings point toward the explanation for an altered hedonic state, improper pleasure evaluation, and dysfunctional reward center processing [37].

Role of DTI in CRPS

In a study by Hotta et al. [38], white matter changes in CRPS was studied using DTI by monitoring the axial diffusivity (AD), mean diffusivity (MD), radial diffusivity (RD), and the correlation of these factors with the motor symptom severity index (MSSI). MSSI is an index created for the study that encompasses factors such as movement-related pain, upper limb disability, hand dexterity, total active range of write movement, and grip strength assessed by a skilled physiotherapist. MD increases were widespread across the white matter skeleton, whereas AD increases were more localized to areas such as the left hemisphere. Corpus callosum and corona radiata showed statistically significant differences in MD, AD, and RD between CRPS patients and healthy controls. It is to be noted that an increase in AD and RD together has been implicated in several neurological diseases such as multiple sclerosis [39], Alzheimer’s disease [40], and amyotrophic lateral sclerosis [41]. MD increase can be indicative of vasogenic edema and neuroinflammation, which is a crucial part of CRPS pathophysiology. There was also a positive correlation noted between motor disability and whole-brain skeletal AD, possibly showing that a high AD (which is usually seen in neurodegenerative conditions) is associated with a worse motor skillset in the patient [38].

In another study by Im et al. about the spectrum of white matter changes observed in CRPS, they concluded that fractional anisotropy (FA), which is a direct indicator of microstructural integrity of white matter, showed a reduction in patients with CRPS when compared to patients without CRPS. Reduced AD was also observed in patients with CRPS. The mean FA (specifically in the prefrontal cortex) values had a negative correlation with the level of pain in patients with CRPS. The reduction of FA was primarily observed in the primary motor and somatosensory cortices of the frontal lobe, specifically in the tracts of the corpus callosum, corona radiata, external capsule, and superior longitudinal fasciculus [42].

Role of skeletal muscle MRI in CRPS

In a series of cases by Nishida et al. [43], it was observed that patients with CRPS had a spectrum of skeletal muscle changes evident on MRI depending on the chronicity of the disease. Patients with chronic CRPS had changes such as atrophy, fibrosis, and fatty infiltration, but newly diagnosed patients had elevated metabolic activity, which was evident on 31-P nuclear magnetic resonance (NMR) spectroscopy. All the patients showed hyperenhancement on T2WI, which is indicative of edema and hyperpermeability of capillaries. The findings during acute CRPS phases are similar to those seen in inflammatory myositis, which might be pointing toward the inflammatory pathophysiology of CRPS wherein microangiopathy might be the culprit for such changes. It might also stem from sympathetic neuronal alterations resulting in abnormal vasoactive substance release, which is also a common observation in CRPS [43].

Role of TPBS in CRPS

In a study by Cheon et al. [44] in which they tried to understand how TPBS can be used to identify and stratify CRPS, it was observed that patients with the disease have different findings when compared to healthy subjects. Establishing criteria for diagnosing CRPS with the help of TPBS was also a part of the study. When there was more uptake in the affected limb than the unaffected side, it was labeled as *I*. When the uptake was equal on both sides, it was labeled as *S*, and when the uptake on the unaffected side was more than the affected side, it was labeled as *D*. Patterns across the three phases of imaging were studied in the aforementioned designated scenarios. The most common pattern observed was D-D-D (reduced blood flow to the affected side in every phase) followed by S-S-S (symmetrical reduction in flow) and S-S-I (initially symmetrical reduction followed by an increase in flow to the affected side in phase 3) [44]. This indicates that patients who have had CRPS for more than a year have significantly reduced blood flow to the affected limb, which is in line with the sympathetic dysregulation that is a part of the pathophysiology of CRPS. This also proves that TPBS can be a very good diagnostic tool for CRPS and can yield significant results.

## Conclusions

Upon a scanning review of various articles and ideas, it can be concluded that CRPS does show a wide spectrum of changes in imaging. There is a significant alteration in cerebral neuronal circuitry due to exposure to chronic pain that is associated with changes in perception of a wide range of aspects, including emotions and their ability to comprehend. As studied in the past, sympathetic dysregulation also plays an important role in the pathophysiology of CRPS, and some changes in imaging (especially muscle and bone imaging) have shown alterations supporting this evidence. There is also a significant role of inflammatory processes in CRPS, which is the reason why some imaging findings are in line with acute or chronic inflammatory processes depending on the duration of symptoms. This supports the idea that imaging can be used to diagnose and monitor CRPS, but there is definitely a need to establish radiological diagnostic criteria to standardize this approach and there is also a requirement to check whether these findings are consistently seen in all the patients or if there are any variations.
